# Chronic Digoxin Toxicity: An Evaluation of Digoxin-Specific Antibodies and Other Management Options

**DOI:** 10.7759/cureus.38692

**Published:** 2023-05-08

**Authors:** Liam M Clifford, William Meere

**Affiliations:** 1 Cardiology, Gosford Hospital, Gosford, AUS

**Keywords:** hypokalemia, isoprenaline, digoxin, digifab, bradycardia, atrial fibrillation

## Abstract

Chronic digoxin toxicity comprises the bulk of digoxin poisonings and can be more difficult to manage than acute intoxications. A 60-year-old lady presented with severe chronic digoxin toxicity after ingesting digoxin 250mcg twice a day (BD) for two weeks. Due to hemodynamic instability on presentation, she was treated with digoxin-specific antibodies and admitted to the coronary care unit. This case of chronic digoxin toxicity did not respond to digoxin-specific antibodies and required intensive cardiac therapy with isoprenaline and intravenous electrolyte replacement, highlighting the complexities in the management of toxicity. Our patient has since recovered and remains stable. There are newer, novel therapies being trialed for the treatment of digoxin toxicity, including dextrose-insulin infusions, therapeutic plasma exchange, and rifampicin, but these require more research and investigation in this cohort of patients.

## Introduction

Digoxin is commonly used in the management of atrial fibrillation (AF) and heart failure. Toxicity involving digoxin can cause conduction abnormalities; however, the differential diagnosis is vast and includes slowed atrioventricular nodal conduction, infiltrative pathologies, senile degeneration, and hypo/hyperkalemia [1-2]. An important distinction is acute versus chronic toxicity, as these two entities require a different management approach. Chronic digoxin toxicity involves the gradual accumulation of digoxin to supratherapeutic levels, and there are several predisposing factors for this, including impaired renal function, advanced age, advanced co-morbidities, and medications [3]. The use of digoxin-specific antibodies in the management of digoxin toxicity, whether it is acute or chronic, doesn't have great evidence from clinical trials in terms of improving mortality. There are limitations to this drug, including the chronicity of the toxicity as well as the hemodynamic instability of the patient [2-4]. We present a case of a patient that presented with chronic digoxin toxicity and didn't respond to digoxin-specific antibodies. They required an isoprenaline infusion with intravenous electrolytes for hemodynamic stability and this demonstrates the current limitations in the management of these difficult cases.

## Case presentation

A 60-year-old lady presented with nausea, vomiting, diarrhea, peripheral visual changes, presyncope, and lethargy after mistakenly consuming digoxin 250mcg twice daily (BD) for two weeks. The patient’s background included paroxysmal AF with prior pulmonary vein isolation, ischaemic heart disease with coronary artery bypass grafting, and mitral valve repair for primary regurgitation. Her latest ejection fraction (EF) was 45%. Other conditions included Hashimoto’s thyroiditis, gastro-esophageal reflux, dyslipidemia, hepatic steatosis, and obesity. Her regular medications were amiodarone 200mg once daily (OD), bisoprolol 5mg OD, digoxin 62.5mcg OD, rivaroxaban 20mg OD, furosemide 40mg OD, spironolactone 12.5mg OD, atorvastatin 40mg OD, perindopril 10mg OD, pantoprazole 40mg OD, and thyroxine 200mcg OD.

**Figure 1:**
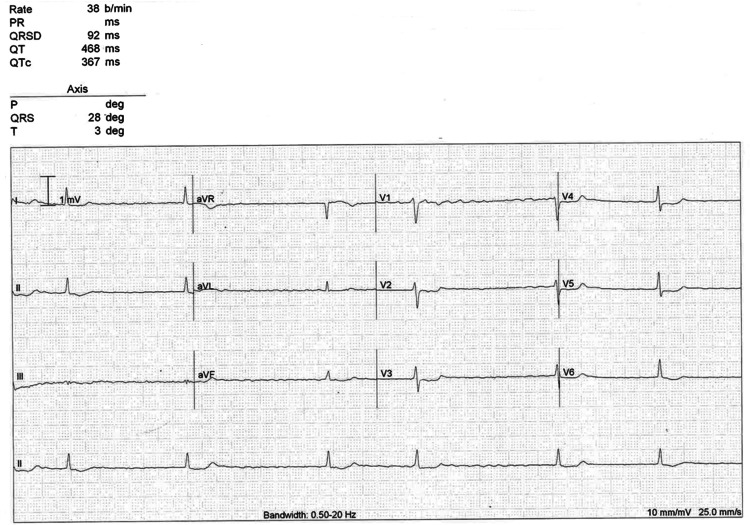
ECG at baseline shows slow atrial fibrillation with minor ST changes.

**Figure 2:**
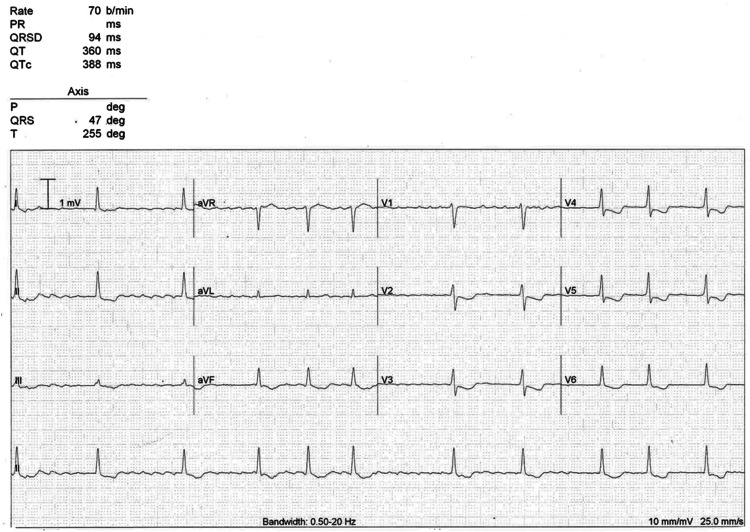
Repeat ECG on day one showing new global ST depression.

**Figure 3:**
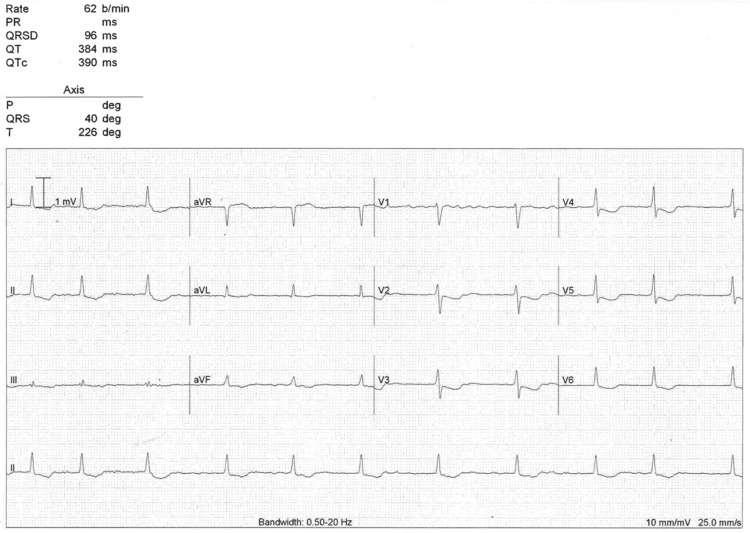
Repeat ECG on day two showing persisting global ST depression.

On presentation to the Emergency Department, her pulse was irregularly irregular, at 25 beats per minute (bpm). The blood pressure was 108/68 mmHg, respiratory rate was 17 breaths per minute, oxygen saturation 97% on room air, and temperature 37.3 °C. Physical examination was unrevealing, and she had a normal chest X-ray. An initial electrocardiogram (ECG) (Figure 1) demonstrated AF, with slow irregular narrow QRS complexes and global downsloping ST depression, which had not been seen on historical ECGs (Figures 2-3). Due to hemodynamic instability, our patient received 40mg of digoxin immune Fab (the local digoxin-specific antibody DigiFab®) intravenously. Her digoxin, bisoprolol, furosemide, and spironolactone were ceased. Despite this, she remained bradycardic and hypotensive, and three hours later received another 40mg of DigiFab®. Owing to minimal effect, atropine 300mcg was administered and isoprenaline was commenced at a rate of 3mcg/hour, with oral and intravenous electrolyte replacement (which included magnesium and potassium) that continued daily.

A transthoracic echocardiogram revealed an EF of 40% with a mildly dilated left ventricle and mild biatrial enlargement. The serum potassium level was 4.8mmol/L (normal range {NR} 3.5-5.2mmol/L), the magnesium level was 0.84mmol/L (NR 0.7-1.2mmol/L), and there was normal renal function. Full blood count, coagulation profile, and liver function tests were normal. To note, the serum digoxin level was 5.2µg/L (NR 0.5-2µg/L). The isoprenaline infusion was slowly weaned after twenty-four hours, with a repeat serum digoxin level of 4.7µg/L. A repeat physical examination revealed mild pitting edema peripherally, so furosemide was recommenced. With stabilized hemodynamics and an improved resting heart rate of 60bpm, our patient was discharged home 24 hours later for outpatient monitoring, with a serum digoxin level down to 1.1µg/L. Our patient was reviewed weekly by a General Practitioner after discharge and attended a weekly outpatient cardiology clinic for an ECG, fluid status, and electrolyte review. After six weeks, her symptoms had resolved, and her digoxin level was within normal limits. She remained in AF, so bisoprolol 5mg mane was recommenced, but digoxin remained ceased, and amiodarone was not commenced due to rate control being achieved with bisoprolol alone.

## Discussion

Digoxin remains a common anti-arrhythmic for the management of AF and severe heart failure. It blocks the sodium-potassium ATPase pump to increase intracellular sodium. This increases the resting membrane potential and subsequently reduces calcium efflux, providing a higher intracellular concentration available to bind troponin-C and thereby increases inotropy [1-3]. Higher calcium also increases automaticity in most cardiac cells which increases the risk of arrhythmia, exaggerated during toxicity. By modulating varied afferent and efferent pathways in the autonomic nervous system, digoxin increases vagal tone, thereby slowing conduction velocity through the AV node and increasing its effective refractory period [1-2].

Chronic digoxin toxicity is the gradual systemic accumulation of digoxin secondary to renal or hepatic dysfunction, or drug interaction, and manifests with supratherapeutic levels over a longer time course and is characterized by higher intracellular levels. While acute toxicity occurs typically within hours, chronic toxicity occurs within days to weeks, and these patients tend to present due to an insidious onset of the symptoms [3]. Risk factors for accumulation include impaired renal function, electrolyte disturbances (particularly hypokalemia and hypomagnesemia as they inhibit tubular secretion of digoxin), advanced age, chronic pulmonary disease, cardiac disease, medications, or changes in dosing [3-4]. Drug-drug interactions are also important, as digoxin competes for the action of P-glycoprotein for excretion and is metabolized by the cytochrome P450 system [5]. Common and important medications that can have this effect include diuretics. Our patient was taking both spironolactone and furosemide for fluid management, contributing to digoxin accumulation.

Digoxin toxicity is divided into cardiac and extracardiac categories, with cardiac manifestations including atrial brady- or tachyarrhythmias and associated hemodynamic instability. This often presents as presyncope or syncope. Common extracardiac symptoms include neurological manifestations (lethargy, delusion, weakness), visual changes (color alterations, scotomas), and gastrointestinal manifestations [1]. Importantly, gastrointestinal symptoms are less pronounced in chronic toxicity, whilst neurological manifestations are typically more common [3]. Severe toxicity may produce refractory hyperkalemia with increased mortality. Possible mechanisms include decompensated heart failure and impaired renal function, or complete body inhibition of the sodium-potassium ATPase pump [4]. The introduction of DigiFab® has reduced the mortality from digoxin toxicity from 20-30% to 5-8%, though it is likely other factors explain this reduction as no clinical trials have clearly established a clinical benefit from this drug [6]. DigiFab® works by binding digoxin molecules to restore ATPase activity [6]. Current indications for administration include a life-threatening arrhythmia, cardiac arrest, hyperkalemia with serum potassium level greater than 5mmol/L, end-organ dysfunction, or a serum digoxin level of 10ng/mL (acute toxicity), or 6ng/mL (chronic toxicity) [1,5-7]. DigiFab® has important limitations, including cost (approximately US$750 per vial of 40mg), limited shelf life, and adverse reactions including exacerbations of heart failure, allergic reactions, and hypokalemia [2].

The management of digoxin toxicity, be it acute or chronic, depends on the underlying arrhythmia. In bradyarrhythmias, initial management should involve DigiFab® and consideration of atropine, particularly if there is hemodynamic instability, although this is less successful in chronic toxicity [1-4,6]. For bradycardic patients who do not respond to DigiFab® or remain hemodynamically unstable, isoprenaline may be considered. It is generally avoided, however, due to the increased risk of malignant ventricular ectopy and the possibility of worsening hypokalemia [4,5,7]. Patients require stringent electrolyte replacement and cessation of causative medications. Temporary pacing may be required, although it is associated with an increased risk of ventricular fibrillation and mortality and should thus be avoided unless all other options have been unsuccessful [2]. The role of gastrointestinal decontamination with charcoal or cholestyramine has very limited evidence in chronic toxicity and is not recommended unless anuric renal failure is present or DigiFab® is unavailable [3-5,7]. There are new approaches to the management of acute digoxin toxicity, with unfortunately no consensus regarding their role in chronic toxicity. Insulin inhibits digoxin activity through a direct effect on the sodium-potassium-ATPase pump, and the successful use of insulin-dextrose infusions has been reported [8-9]. Therapeutic plasma exchange has been trialed with some success, mainly in the context of renal failure, although it is unable to remove free digoxin. Additionally, the P-450 inducer rifampicin has been trialed. It reduces digoxin half-life, and there have been reports of limited successful treatment [10]. Unfortunately, despite the renal excretion of digoxin, the use of hemodialysis for elimination has not proven effective, especially in chronic toxicity, given the large tissue distribution of the drug in a steady state [5].

## Conclusions

This case demonstrates the complexities associated with the management of chronic digoxin toxicity. Owing to the high cellular levels of digoxin in chronic toxicity, DigiFab® has demonstrated limited efficacy, and further measures were warranted to enhance intrinsic conduction. There are several novel and core targeted therapies, including dextrose-insulin infusions, therapeutic plasma exchange, and rifampicin. These appear to hold promise as rescue options when DigiFab® has failed, though they require further rigorous investigation.
